# Chironomid dataset from Mutterbergersee: A late-Holocene paleotemperature proxy record for the Central Eastern Alps, Austria

**DOI:** 10.1016/j.dib.2022.108431

**Published:** 2022-07-01

**Authors:** Elena A. Ilyashuk, Oliver Heiri, Wojciech Tylmann, Boris P. Ilyashuk

**Affiliations:** a Department of Ecology, University of Innsbruck, Technikerstrasse 25, Innsbruck 6020, Austria; b Department of Environmental Sciences, Geoecology, University of Basel, Klingelbergstrasse 27, Basel 4056, Switzerland; c Faculty of Oceanography and Geography, University of Gdańsk, Bażyńskiego 4, Gdańsk 80309,Poland

**Keywords:** European Alps, Little Ice Age, Lake sediments, Chironomidae, Subfossils, Summer air temperature, Transfer function approach

## Abstract

We present a dataset of subfossil chironomid assemblages in the MUT-10 sediment core obtained from the high alpine lake Mutterbergersee in the Austrian Alps in 2010. The data were presented in the research article by Ilyashuk et al. (2019) “The Little Ice Age signature in a 700-year high-resolution chironomid record of summer temperatures in the Central Eastern Alps”. In addition to the results of the chironomid analysis of 100 sediment samples presented in this article, we also include chironomid assemblage data from an additional 48 sediment samples that complement this dataset. The data includes raw chironomid counts, percent abundance of chironomid taxa, as well as mean July air temperature estimates derived from the chironomid record based on a chironomid-temperature transfer function. We also provide information on age-dating of the sedimentary sequence. Given the high temporal resolution and the robust age-depth model of the record, the chironomid-based reconstruction of temperature since AD 1300 provides a detailed documentation of climate change in the Eastern Alps from the Little Ice Age onwards and can be used for comparison with other independent proxy-based climate reconstructions. In addition to the data, we detail the sample processing for subfossil chironomid analysis and provide a detailed description of the reconstruction technique used for producing chironomid-based quantitative temperature inferences.

## Specifications Table

**Table:** 

Subject	Earth and Planetary Sciences
Specific subject area	Paleolimnology, paleoecology, paleoclimate
Type of data	TablesFigure
How the data were acquired	A sediment core (MUT-10) was acquired from the deepest part of the lake with a UWITEC gravity corer USC 06000. The core was sectioned contiguously using a UWITEC core cutter at increments of 0.22 cm. Chronological control was based on ^210^Pb activity determinations and accelerator mass spectrometry (AMS) ^14^C dates derived from terrestrial plant macrofossils. The OxCal software was used for constructing age-depth relationships from these chronological assessments. Sediments were treated with heated KOH and sieved through a 100 μm mesh sieve. Head capsules of subfossil chironomids were manually sorted and picked out from the sieve residue in a Bogorov counting tray under a stereomicroscope (ZEISS Stemi 2000) at 20–40 × magnification, and permanently mounted on microscope slides in Euparal® (Carl Roth Gmbh) mounting medium. Identification of chironomid taxa was carried out with a compound microscope (Optika B-600Ti) at 200–400 × magnification. Mean July air temperatures were reconstructed using a transfer function based on a combined Swiss and Norwegian chironomid–temperature calibration data-set with the software package C2.
Data format	RawAnalyzed and calculated
Description of data collection	The age-depth model indicates that MUT-10 sediment core (32.6 cm long) spans the past ∼700 years. The chironomid data set comprises 18,254 chironomid remains recovered from 148 core samples at a vertical resolution of 0.22 cm. A minimum of 100 chironomid head capsules (range: 100–209) was hand-picked and taxonomically identified in each sample. Twenty-three chironomid taxa were identified in the sediment sequence. Quantitative temperature estimates were produced with a chironomid–temperature transfer function based on a Weighted Averaging – Partial Least Squares (WA-PLS) approach.
Data source location	• Department of Ecology, University of Innsbruck • Innsbruck, Austria • Coring location for MUT-10 core: 47°01′01″ N, 11°07′41″E
Data accessibility	Repository name: Mendeley DataData identification number: 10.17632/nrchz3wc88.1Direct link to the data: https://data.mendeley.com/datasets/nrchz3wc88/1
Related research article	E.A. Ilyashuk, O. Heiri, B.P. Ilyashuk, K. Koinig, R. Psenner, The Little Ice Age signature in a 700-year high-resolution chironomid record of summer temperatures in the Central Eastern Alps, Clim. Dyn. 52 (2019) 6953–6967. 10.1007/s00382-018-4555-y[1].

**Value of the Data**

• Our data provide a continuous and exceptionally highly (∼4.8 yrs) resolved chironomid record and associated chironomid-inferred summer temperature estimates for the late Holocene (AD 1300‒2010) from a high-alpine lake in the Central Eastern Alps, Austria.
• These data capture changes in species composition and assemblage structure of chironomids and provide a unique opportunity for detailed investigation of lake system responses to climate change from the early years of the Little Ice Age (LIA), the coldest period of the last millennia in Europe, to the current warm period.
• The high-resolution 700-year long chironomid-based mean July air temperature reconstruction from the remote mountain lake allows insights into the climatic deterioration during the LIA and recent climate change in the Alpine region.
• The temperature reconstruction can be used for comparison with other independent regional or synoptic proxy-based climate reconstructions on multi-annual and longer timescales.
• Our data are of particular interest for researchers involved in studying effects of climate change on ecosystems as well as in simulating and reconstructing past climate conditions.

## 1 Data Description

**Fig. 1:**
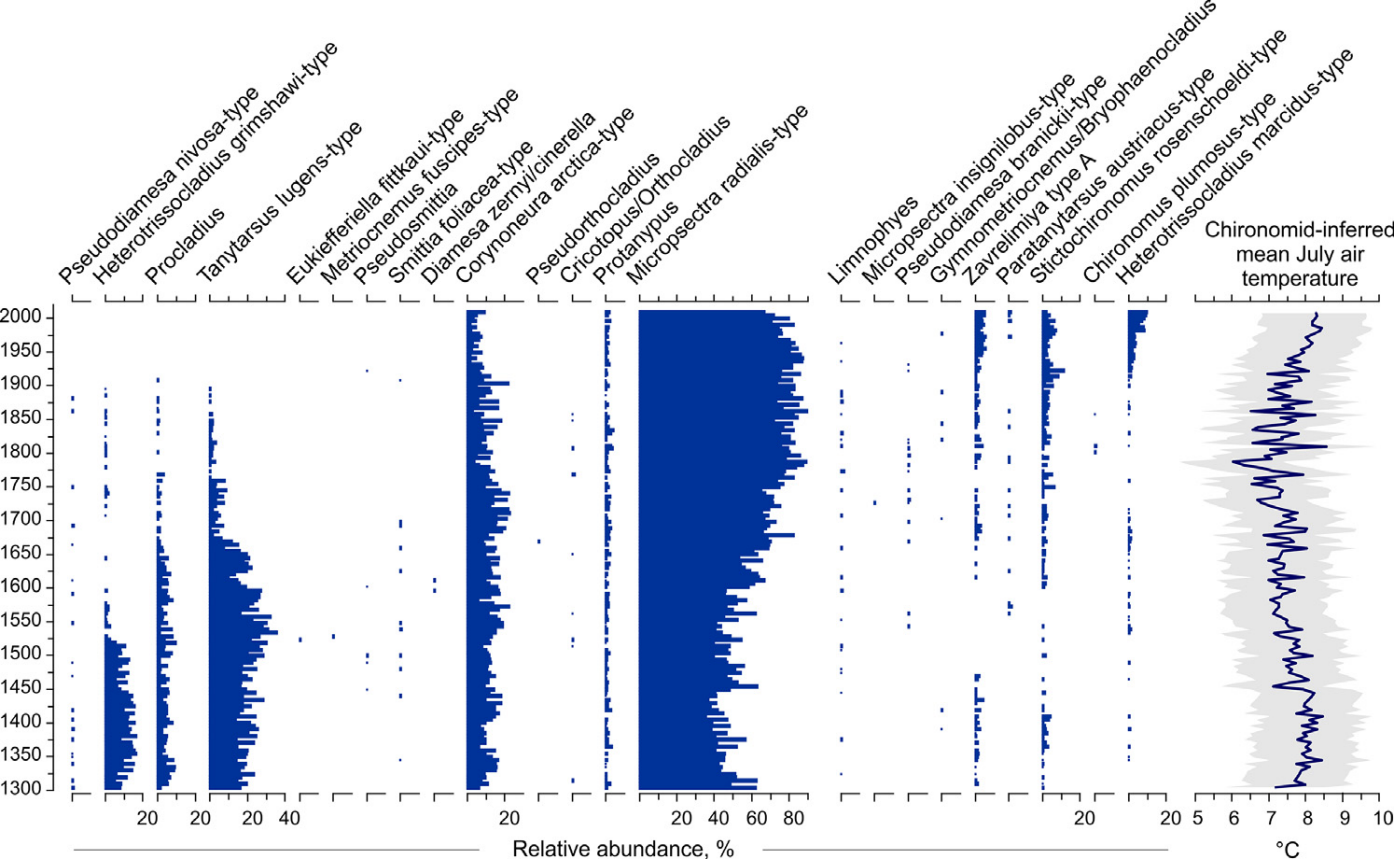
Subfossil chironomid stratigraphy of all taxa recorded in the MUT-10 sediment sequence from Mutterbergersee and chironomid-inferred mean July air temperature plotted together with estimated sample-specific errors of prediction (colored envelope).

Data reported herein have been derived from the investigation of long-term (AD 1300–2010) changes in chironomid (non-biting midges, Insecta: Diptera: Chironomidae) assemblages in sediment core MUT-10, retrieved from Mutterbergersee (MUT), a high-alpine lake in the Central Eastern Alps (Stubai Alps, Tyrol). A total of 148 sediment samples of 0.22 cm thickness were taken from a 32.6 cm sediment core. Subfossil chironomid analysis was performed on 100 sediment samples (every sample at the top 11.5 cm of the core and every other one at the lower part) as reported in Ilyashuk et al. [1,2]. In addition we report analyzes from 48 additional samples carried out to increase the temporal resolution of and provide additional detail for our quantitative chironomid-based temperature reconstruction. Full chironomid data and paleotemperature estimates from the complete MUT-10 sequence consisting of all available 148 samples are presented at Fig. 1. Twenty-three chironomid taxa were identified in the sediment sequence.

The dataset is deposited at Mendeley Data (https://data.mendeley.com/datasets/nrchz3wc88/1). The data are provided in five separate Excel sheets. The first sheet (metadata) provides information about the coring site, archive type and related research articles. The second one (raw chironomid count) contains subfossil chironomid head capsule count data (= number of head capsules recovered per sample). The third one (chironomid percentages) contains the relative abundances of each taxon calculated with respect to the total chironomid remains enumerated per sample. The fourth sheet (chironomid-inferred T_July) includes mean July air temperature data calculated from the chironomid assemblages using a Swiss-Norwegian chironomid–temperature inference model [3]. Moreover, the chronological framework data are available in the fifth sheet (dating) containing information on the ^210^Pb and ^14^C measurements in sediment core MUT-10.

## 2 Experimental Design, Materials and Methods

### 2.1 Study Site, Sampling and Chronology

Mutterbergersee (MUT), a typical, oligotrophic mountain lake, is located above the treeline at 2483 m a.s.l. in the Stubai Alps. This small lake with a water surface area of 3.8 ha and a maximum depth of 8.0 m lacks well-developed inflows and outflows and has continuous sedimentation of fine material in the deepest part. The MUT catchment is glacier-free and covers ∼20.0 ha in area. The lake is remote, has no permanent settlements in the immediate vicinity and is almost undisturbed by direct human activities. Such small alpine lakes are of particular interest for paleoclimatic studies, because they, being closely coupled with atmospheric forcing factors, are sensitive to climate change, but can also integrate response signals over time and provide a variety of proxy climate indicators [4].

Sediment core MUT-10 (32.6 cm long) was retrieved at the deepest point (8 m) of the lake (47°01′01″ N, 11°07′41″E) in September 2010 using a UWITEC (an Austrian engineering company) gravity corer USC 06000 equipped with an automatic released ball core catcher and fitted with standard PVC tubes (ID/OD 59.5/63 mm). To achieve high temporal resolution, the core was sectioned contiguously using a UWITEC core cutter and space laminae of 0.22 cm thick. All samples were packed in Thermo Scientific™ Samco™ containers and kept under refrigeration (4 °C). A total of 148 samples were obtained from the core.

The age-depth model of the MUT-10 record is based on sixteen ^210^Pb activity measurements (down to 6.7 cm depth) and three accelerator mass spectrometry (AMS) radiocarbon dates derived from terrestrial plant macrofossils for the deeper section of the core. ^210^Pb activity concentration in recent sediments was determined by alpha-spectrometry at the University of Gdańsk, Poland and the ^210^Pb chronology was based on the constant rate of supply (CRS) dating model [5]. AMS radiocarbon dating was carried out at the Poznań Radiocarbon Laboratory (Poland) and the Beta Analytic Radiocarbon Dating Laboratory (Miami, FL, USA). Age-depth modeling was performed on-line using the OxCal 4.3.2 Bayesian Chronological Modeling software [6], which integrates the mid-latitude Northern Hemisphere appropriate IntCal13 calibration curve [7]. According to the age-depth model, an average temporal resolution is ∼4.8 years per sample in the sediment sequence. For a more detailed explanation of the establishment of the chronology, we refer the reader to the original research articles [1,2].

### 2.2 Chironomid Analysis and Temperature Reconstruction

Sample processing for subfossil chironomid analysis included deflocculating in heated 5% KOH and sieving through a 100 μm mesh [8]. Head capsules of subfossil chironomids were sorted from the residue in a Bogorov counting tray under a stereomicroscope (ZEISS Stemi 2000) at 20–40 × magnification. Afterwards head capsules were dehydrated in 100% ethanol and permanently mounted ventral side up on microscope slides in Euparal® (Carl Roth Gmbh) mounting medium for identification. Chironomids were identified under a compound microscope (Optika B-600Ti) at 200–400 × magnification. Chironomid taxonomy followed Brooks et al. [9] and Andersen et al. [10]. A minimum of 100 chironomid head capsules were counted and identified in each sample. Depending on the chironomid abundance, aliquots of 1–6 g of wet sediment were processed. The chironomid percentage stratigraphic diagram for MUT was plotted with C2 software [11].

Quantitative mean July air temperature estimates from MUT were produced by applying a combined Swiss-Norwegian chironomid–temperature calibration data-set and inference model based on chironomid assemblage data from 274 lakes spanning a July air temperature gradient of 3.5–18.4 °C [3]. Reconstructions were based on Weighted Averaging – Partial Least Squares (WA-PLS) regression [12] with two components. The model predicts mean July air temperature within the calibration data set with a cross-validated Root Mean Square Error of Prediction (RMSEP) of 1.40 °C and a cross-validated coefficient of determination (r^2^) between inferred and observed July air temperature values of 0.87. Error statistics, including sample-specific estimated Standard Errors of Prediction (eSEP), were calculated based on bootstrapping (9999 bootstrap cycles). Temperature reconstruction was conducted using the software package C2 [11] and based on square-root transformed percentage data. For reconstruction, 19 sites identified as outliers in the calibration dataset [3] were excluded and the final transfer function was based on the remaining 255 lakes of the calibration dataset.

## CRediT authorship contribution statement

**Elena A. Ilyashuk:** Conceptualization, Methodology, Investigation, Visualization, Project administration, Funding acquisition, Writing – original draft. **Oliver Heiri:** Software, Validation, Formal analysis, Writing – review & editing. **Wojciech Tylmann:** Investigation, Writing – review & editing. **Boris P. Ilyashuk:** Methodology, Investigation, Writing – review & editing.

## Declaration of Competing Interest

The authors declare that they have no known competing financial interests or personal relationships that could have appeared to influence the work reported in this paper.
